# Impact of the SARS-CoV-2 pandemic on the course and treatment of appendicitis in the pediatric population

**DOI:** 10.1038/s41598-021-03409-2

**Published:** 2021-12-14

**Authors:** Alicja Pawelczyk, Malgorzata Kowalska, Marzena Tylicka, Olga Martyna Koper-Lenkiewicz, Marta Diana Komarowska, Adam Hermanowicz, Wojciech Debek, Ewa Matuszczak

**Affiliations:** 1 Pediatric Surgery and Urology Department, Medical University of Bialystok, Waszyngtona 17, 15-724 Bialystok, Poland; 2 Biophysics Department, Medical University of Bialystok, Bialystok, Poland; 3 Department of Laboratory Clinical Diagnostics, Medical University of Bialystok, Bialystok, Poland

**Keywords:** Diseases, Health care, Medical research, Risk factors

## Abstract

SARS-CoV-2 is a highly contagious virus causing mainly respiratory track disease called COVID-19, which dissemination in the whole world in the 2020 has resulted in World Health Organisation (WHO) announcing the pandemic. As a consequence Polish Government made a decision to go into a lockdown in order to secure the population against SARS-CoV-2 outbreak what had its major influence on the Polish Health Care System. All of the social and medical factors caused by the pandemic might influence children’s health care, including urgent cases. The aim of this survey was the analysis of medical charts with focus on the course and results of surgical treatment of children who underwent appendectomy before and during the COVID-19 pandemic. Material and methods: We performed analysis of charts of 365 subjects hospitalized in the Pediatric Surgery Department from 1st January 2019 to 31st December 2020 because of acute appendicitis. Patients were divided into two groups—those treated in 2019—before pandemic outbreak, and those treated in 2020 in the course of pandemic. Results: the most common type of appendicitis was phlegmonous (61% of cases in 2019 and 51% of cases in 2020). Followed by diffuse purulent peritonitis (18% of cases in 2019 vs 31% of cases in 2020), gangrenous (19% of cases in 2019 vs 15% of cases in 2020) and simple superficial appendicitis (1% of cases in 2019 vs 3% of cases in 2020). There was statistically significant difference in the length of hospitalization: in 2019 the mean length of hospi-talization was 4.761 vs 5.634 in 2020. Laparoscopic appendectomy was performed more frequently before the COVID period (63% of cases treated in 2019 vs 61% of cases treated in 2020). In the pandemic year 2020, there was double increase in the number of conversion from the laparoscopic approach to the classic open surgery. In the year 2019 drainage of abdominal cavity was necessary in 22% of patients treated with appendectomy, in 2020 the amount of cases threated with appendectomy and drainage increased to 32%. Conclusions: fear of being infected, the limited availability of appointments at General Practitioners and the new organisation of the medical health care system during pandemic, delay proper diagnosis of appendicitis. Forementioned delay leads to higher number of complicated cases treated with open appendectomy and drainage of abdominal cavity, higher number of conversions from the laparoscopic to classic open technique, and longer hospitalization of children treated with appendectomy in the year of pandemic.

## Introduction

SARS-CoV-2 is a highly contagious virus causing mainly respiratory track disease called COVID-19 (corona-virus-disease-2019), which dissemination in the whole world in the 2020 has resulted in World Health Organization (WHO) announcing the pandemic on the 11th of March 2020^1,2^.

As a consequence Polish Government made a decision to go into a lockdown in order to secure the population against SARS-CoV-2 outbreak what had its major influence on the Polish Health Care System which was adapted to the unusual conditions. Those changes, among others, consisted of reorganizing the structures of hospitals and outpatient clinic such as: modernization of ICU facilities, usage of protective equipment, redistribution of medical staff to covid hospitals and covid units^2^. Even ordinary medical appointments at general practices and outpatient clinics were either delayed or done remotely over the phone or online. Moreover, planned procedures were cancelled, and on many occasions only urgent cases were hospitalized, to focus the medical personnel and hospitals on individuals with covid-19^2,3^.

In addition, apprehension of being infected triggered people to minimize their contact with medical staff, units, and other places with elevated risk of acquiring infection. The delay of an accurate diagnosis and adequate treatment seems to be mainly due to the avoidance of medical care^4^.

In the most situations, symptoms and complications connected with coronavirus infection among pediatric population are less severe than in adults^5^. Nevertheless, all of the social and medical factors caused by the pandemic might influence children’s health care, including urgent cases^5,6^.

One of the most frequent emergency conditions suffered by children is the appendicitis, in which the main symptom is abdominal pain^6,7^. Generally thorough clinical examination made by specialist surgeon is sufficient for the right diagnosis. In such event, appendectomy is a standard and efficient procedure^6,7^.

In our survey we performed analysis of medical charts with focus on the course and results of surgical treatment of children who underwent appendectomy before and during the COVID-19 pandemic, more specifically, the years of 2019 and 2020 in the Pediatric Surgery Department. We compared the influence of this unusual situation on the course and methods of treatment, hospitalization time, and the rate of complications among the children treated with appendectomy.

## Material and methods

We performed analysis of charts of 365 subjects hospitalized in the Pediatric Surgery Department from 1st January 2019 to 31st December 2020, because of acute appendicitis. Patients were divided into two groups—those treated in 2019—before pandemic outbreak, and those treated in 2020 in the course of pandemic. In those two groups, we compared demographic data, laboratory findings, methods of operation, type of appendicitis, the necessity of the drainage of the abdominal cavity, and the length of hospitalization. The inclusion criteria were: patients operated on because of acute appendicitis, the exclusion criteria were: any concomitant therapies or chronic conditions.

In each case, acute appendicitis was diagnosed mainly by clinical examination and additional findings such as laboratory tests, including blood morphology, CRP and abdominal ultrasonography. The diagnosis were confirmed histopathologically as: simple inflamed, phlegmonous and gangrenous.

Appendectomy was performed as an emergency, in 2020 after COVID-19 Antigen Rapid Test which was required for all emergency admissions by the hospital regulations. Furthermore, the type of surgical treatment (classic open or laparoscopic) was chosen by the surgeon based on clinics and findings in imaging studies. We also calculated the percentage of conversions. This survey was approved by Ethics Committee of Medical University of Bialystok, Poland. An exemption on informed consent was obtained from Ethics Committee of Medical University of Bialystok, Poland. All methods were performed in accordance with the relevant guidelines and regulations.

We assumed that the COVID-19 pandemic and the new organization of the medical health system, delayed admissions to the hospitals and widespread fear of getting infected could worsened the course and results of treatment of children with appendicitis.

### Statistical analysis

Statistical analysis was performed using the STATISTICA PL release 12.5 Program. Data normality was analyzed using Shapiro–Wilk test. Results are presented as median (Me) with the 25th and 75th percentiles (IQs). For tested parameters which did not follow the normal distribution the differences between two independent groups (data from years 2019 and 2020) were assessed using the Mann–Whitney U test. To compare means independent samples T-test was used. Differences were considered significant with the value of p < 0.05.

## Results

Medical records of 365 children diagnosed and operated on because of appendicitis in the Pediatric Surgery Department between 2019 and 2020 were evaluated. The group consisted of 215 boys and 150 girls. In the year 2019, before COVID-19 pandemic, 201 children were operated on because of the appendicitis (median age 11.631), 102 boys and 63 girls. In the year 2020 during COVID-19 pandemic, 164 patients were operated on because of the appendicitis (median age 11.023), 113 boys and 87 girls. We noted difference in the time between the onset of symptoms, and presentation to the hospital: in 2019—2% of patients presented within 12 h from the onset of symptoms, 60% within 12–24 h, 20% within 24–48 h and 18% after more than 48 h from the onset of symptoms. In 2020, 3% of patients were admitted to surgery department within 12 h from the onset of symptoms, 50% within 12–24 h from the onset of symptoms, 15% within 24–48 h, and 32% after more than 48 h from the onset of symptoms (the difference was statistically significant). In the year 2020, only about 10% of parents complained that they were unable to schedule appointment with GP, the rest headed straight to the Emergency Department. Parents did not complain about limitations in public transport due to lock down, and we did not note the lack of bed in our hospital.

We also did not notice the difference between 2019 and the pandemic year 2020 in the mean time between diagnosis of appendicitis and the surgery. All patients admitted to our ED had COVID-19 Antigen Rapid Test—among our patients in the pandemic year 2020 only two cases were COVID positive. Also protective measures in surgery preparation for COVID patients were not causing substantial delay in performing appendectomy. There was no statistically significant difference in the BMI of patients: in 2019 the mean BMI was 19.479 (SD = 4.208) vs 18.738 in the year 2020 (SD = 4.391) p = 0.129.

As a rule, the type of acute appendicitis was established according to findings during surgery: non-complicated cases: simple superficial appendicitis, followed by more inflamed appendicitis phlegmonous, then gangrenous without perforation and localized collection of fluid, and complicated cases of diffuse peritonitis caused by perforated necrotic appendix.

**Figure 1:**
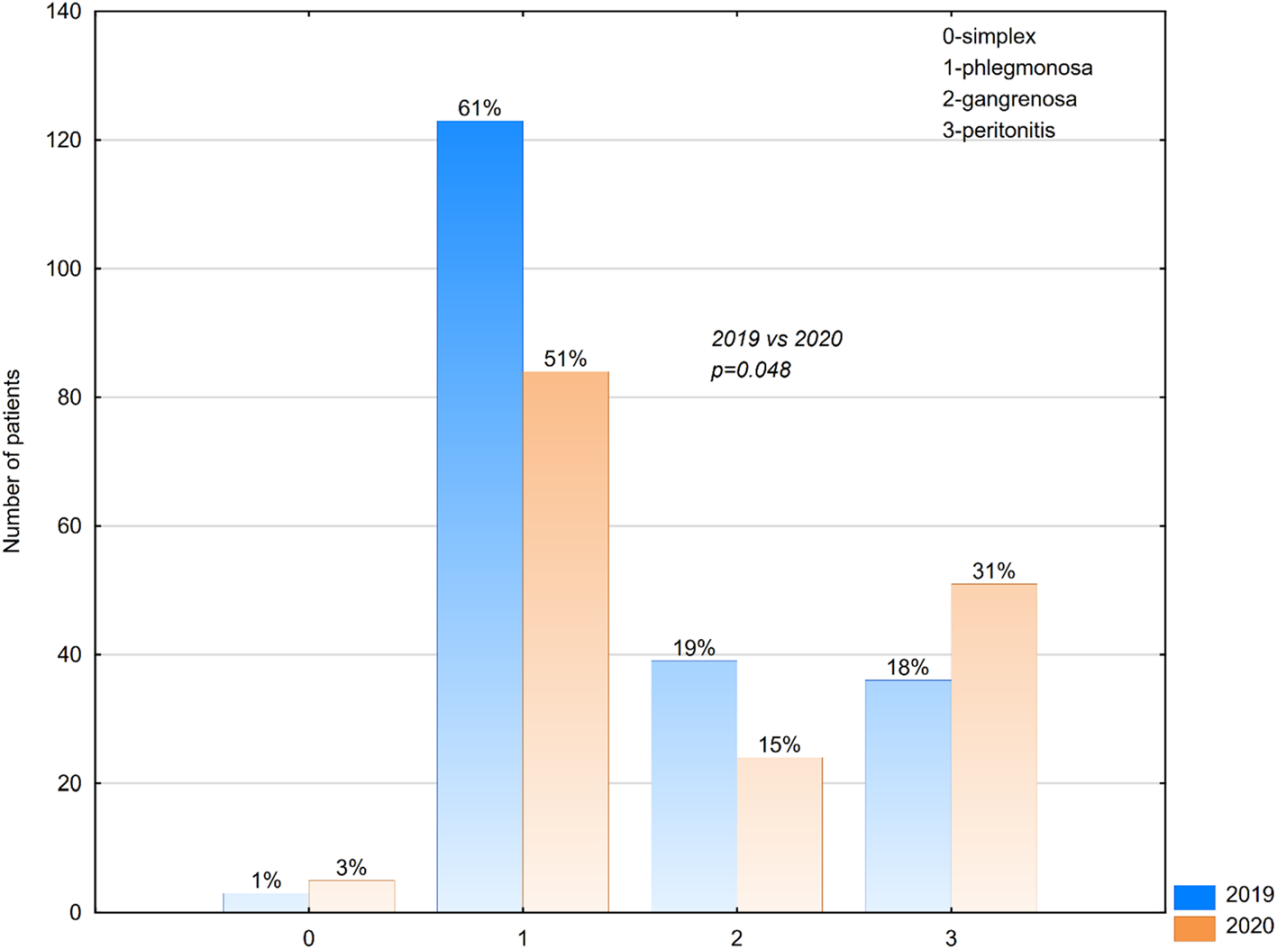
Diagnosis of appendicitis among patients treated in 2019 and 2020.

Generally, the most common type of appendicitis was phlegmonous (61% of cases in 2019 and 51% of cases in 2020). Followed by diffuse purulent peritonitis with perforation of appendix (18% of cases in 2019 vs 31% of cases in 2020), gangrenous (19% of cases in 2019 vs 15% of cases in 2020) and simple superficial appendicitis (1% of cases in 2019 vs 3% of cases in 2020) (Fig. 1). The differences between the number of each type of appendicitis in year 20,219 and 2020 are statistically significant (p = 0,048).

**Figure 2:**
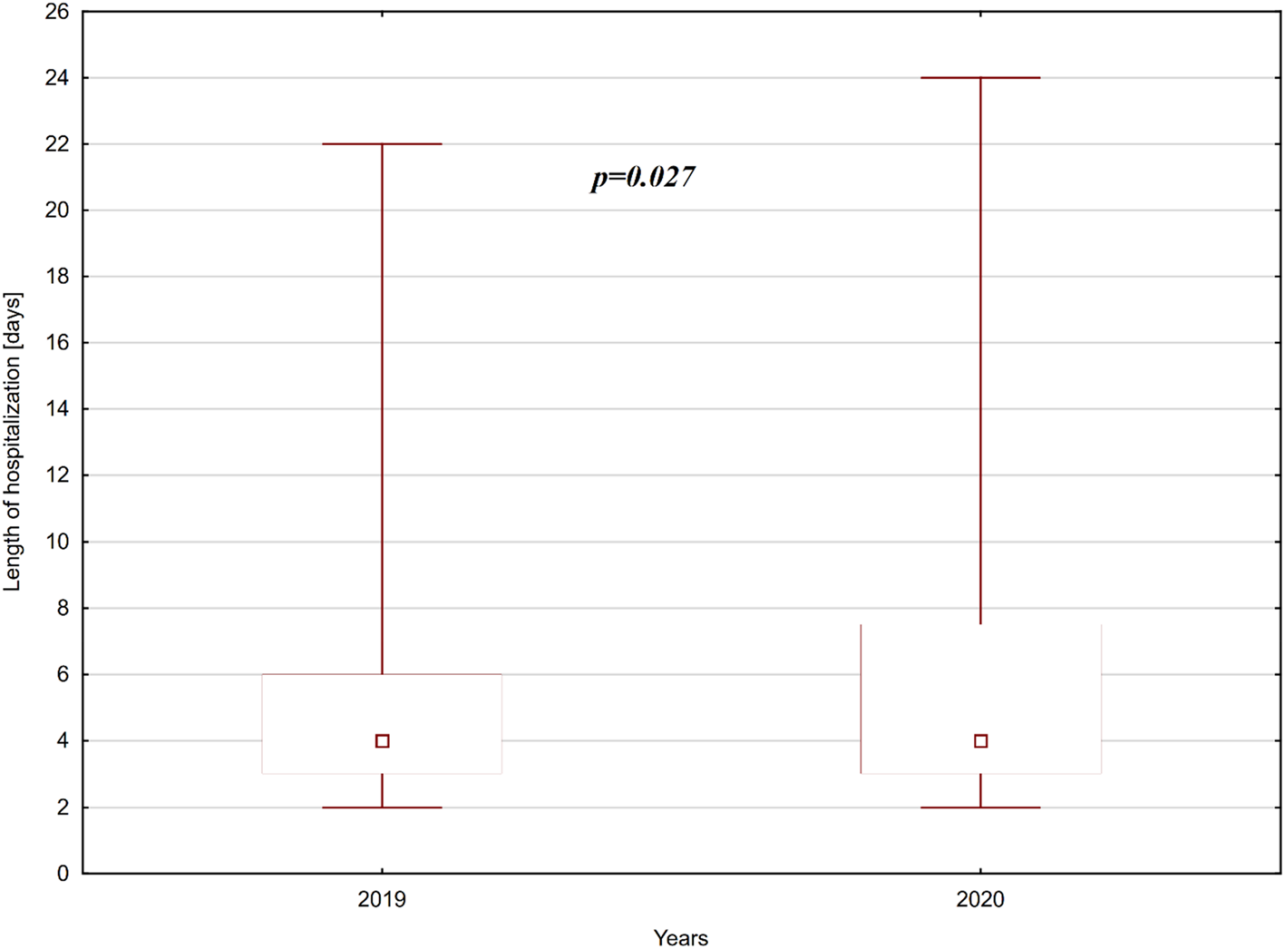
Length of hospitalization among patients treated with appendectomy in 2019 and 2020.

We also observed a statistically significant difference in the length of hospitalization of patients with appendicitis in the year 2019 vs 2020 (p = 0,012). In the year 2019 the mean length of hospitalization was 4.761 ± 0.69 vs 5.634 ± 1.09 in 2020 (Fig. 2).

**Figure 3:**
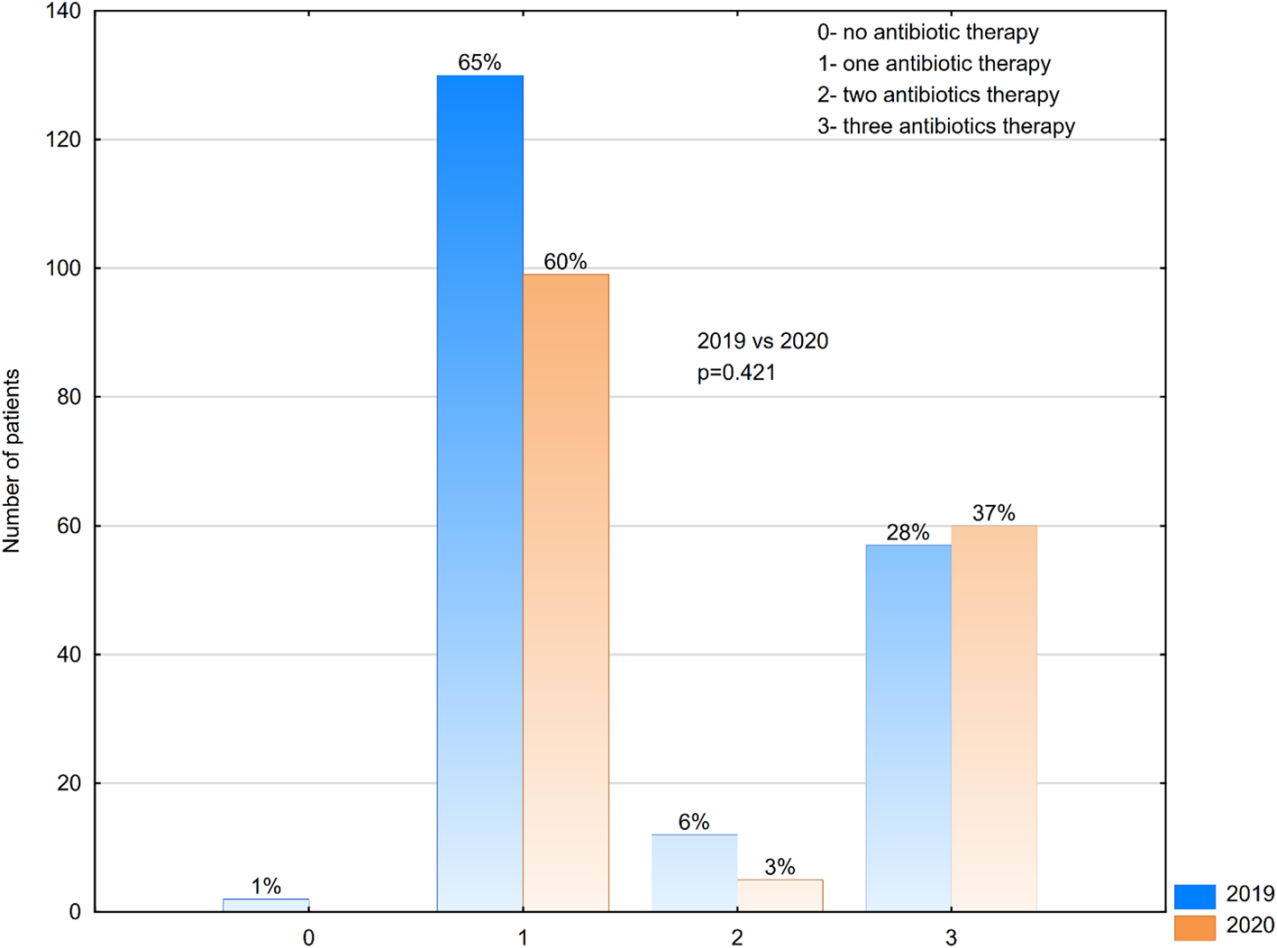
Antibiotic therapy among patients treated with appendectomy in 2019 and 2020.

However there was no statistically significant difference in the antibiotic therapy in patients treated in 2019 vs 2020 (Fig. 3) (p = 0.421). Our standard protocol for antibiotic therapy in patients with acute appendicitis is Cefazolin as a perioperative prophylaxis in non-complicated cases of appendicitis, treatment with two antibiotics—second generation cephalosporin and Metronidazole in cases of appendicitis gangrenous without perforation and localized collection of purulent fluid, and in complicated cases with diffuse peritonitis and perforation of necrotic appendix 3 antibiotics—second generation cephalosporin, Metronidazole and Aminoglycoside.

**Figure 4:**
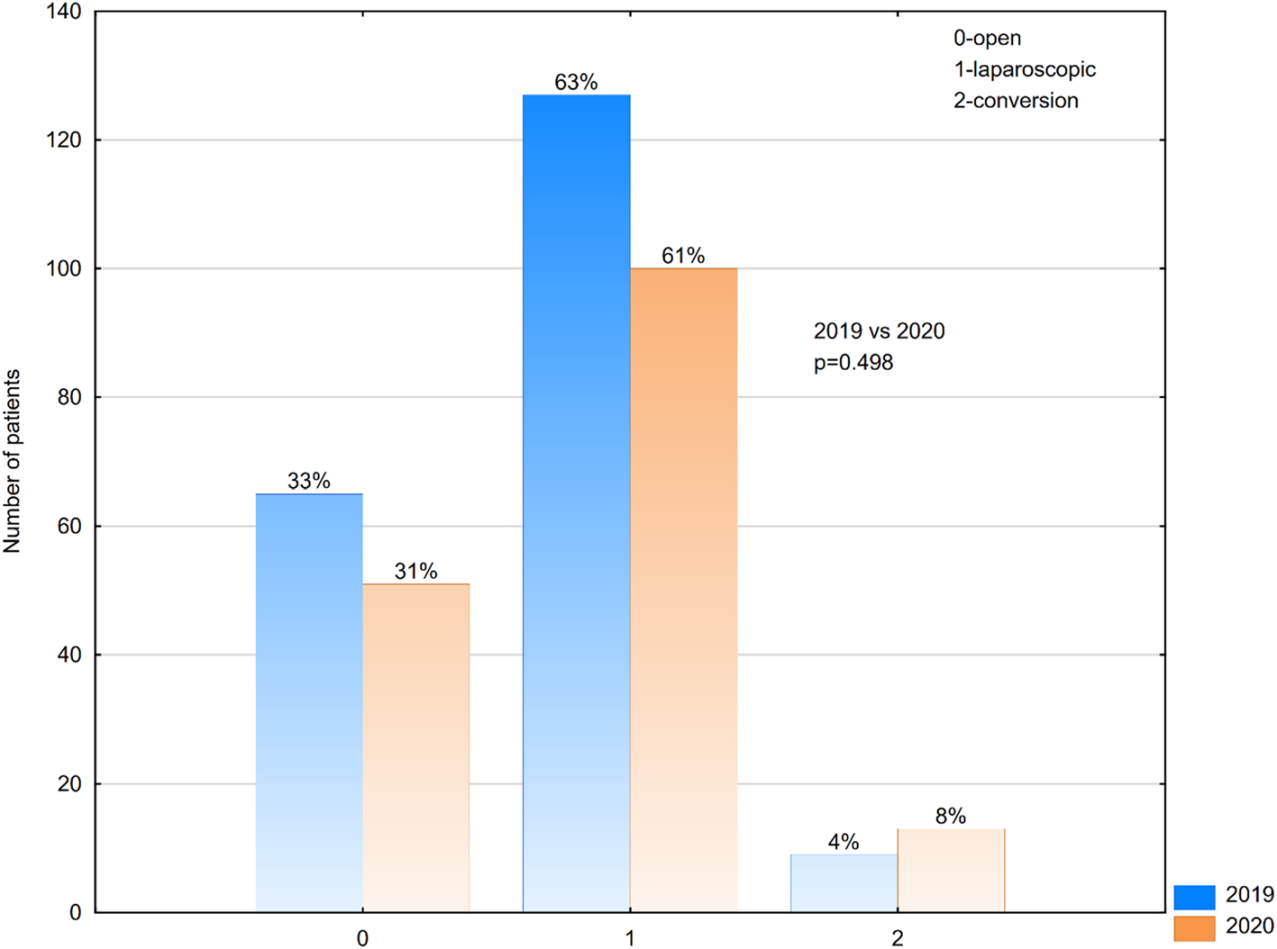
Type of surgical approach among patients treated with appendectomy in 2019 and 2020.

**Figure 5:**
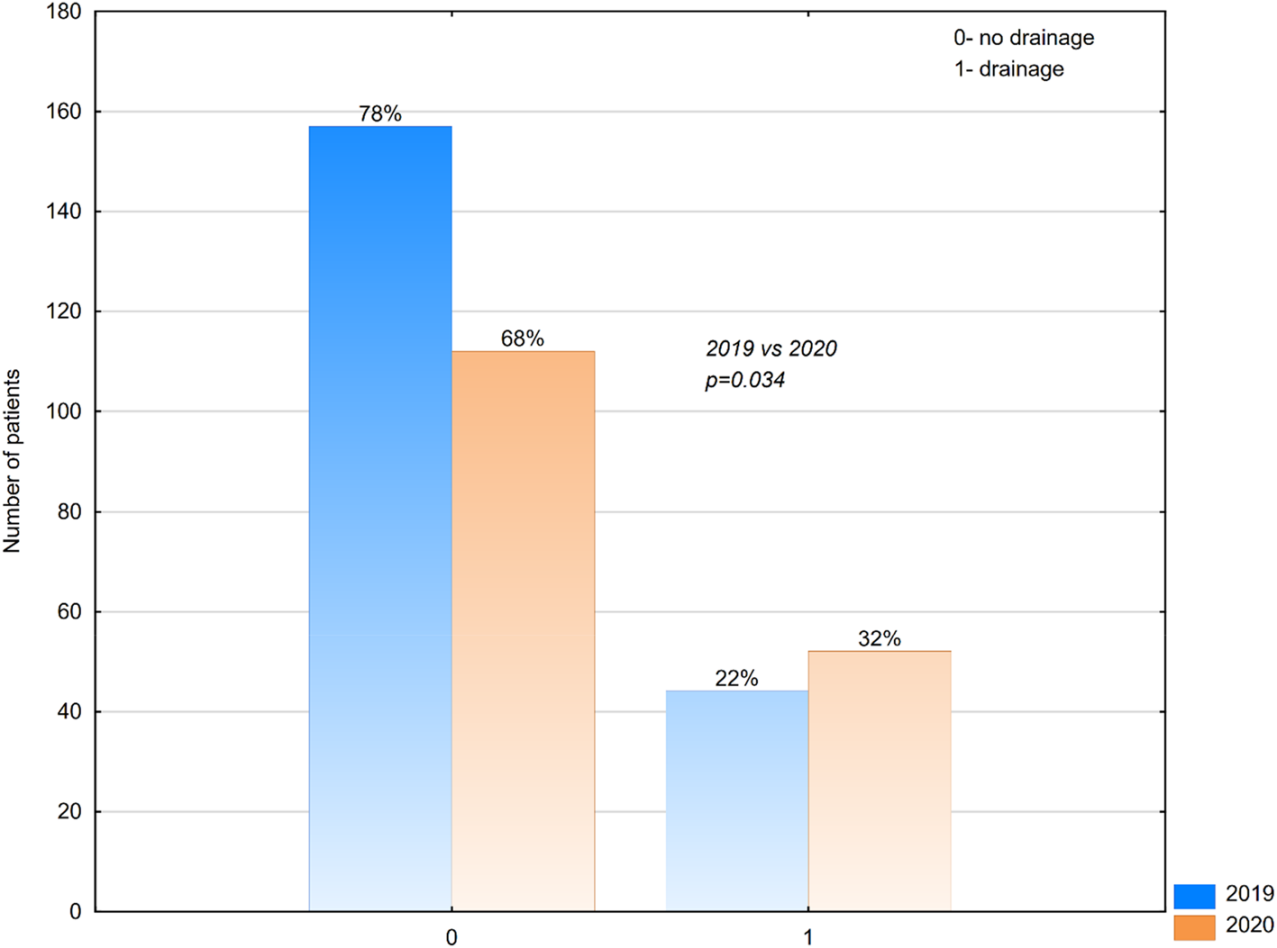
The amount of cases threated with appendectomy and drainage in 2019 and 2020.

During the pandemic year 2020, our department did not change the protocols of treatment of appendicitis. We do not implement conservative treatment of appendicitis. Non-complicated cases of appendicitis were operated on laparoscopically, complicated cases were treated with open appendectomy. Still we noticed divergence in the type of surgical approach between years 2019 and 2020. Laparoscopic appendectomy was performed more frequently before the COVID period (63% of cases treated in 2019 vs 61% of cases treated in 2020). In the pandemic year 2020, there was double increase in the number of conversion from the laparoscopic approach to the classic open surgery—4% of cases in 2019 vs 8% of cases in 2020) (Fig. 4), those cases required conversion to classic open method due to technical difficulty. The difference in the quantity of cases treated with different type of surgical approach was statistically significant (p = 0,498). In the year 2019, 22% of patients required peritoneal drain for continuous drainage after appendectomy. In 2020 the amount of cases threated with appendectomy and drainage increased to 32% (Fig. 5). The difference is statistically significant (p = 0,034).

The differences in the white blood count (p = 0,221) and CRP serum levels (p = 0,283) in our patients with appendicitis treated in the year 2019 vs 2020 were nonsignificant (Tables 1,2).

**Table 1:** WBC concentration in plasma of children treated because of an appendicitis in the years 2019 and 2020.

	WBC concentration concentration [× 103/μL]	
	**2019 **N = 201	**2020 **N = 164
Median	**13.7**	**14.8**
Percentiles (25–75%)	10.66–17.70	11.89–17.20
p-value*	p = 0.221	

*A *p value* < *0.05* is considered to show a significant difference between groups (according to the Mann–Whitney U test).

**Table 2:** CRP concentration in plasma of children treated because of an appendicitis in the years 2019 and 2020.

	CRP concentration [mg/L]	
	2019 N = 201	2020 N = 164
Median	**20.97**	**25.03**
Percentiles (25–75%)	6.72–59.35	7.18–77.86
p-value*	p = 0.283	

*A *p value* < *0.05* is considered to show a significant difference between groups (according to the Mann–Whitney U test).

## Discussion

The COVID-19 pandemic has not only changed our life but also the way medical care is provided. We wanted to assess the influence of COVID-19 pandemic on the treatment of common surgical emergency—the inflammation of the appendix.

When we compared medical charts of pediatric patients treated with appendectomy in 2019 vs 2020 we observed substantial changes. Substantial rise in the number of complicated cases in children with appendicitis in 2020, caused more frequent application of drainage of the peritoneal cavity. We speculate that worse outcomes in the year of pandemic, was caused by the delay of surgical treatment, due to fear of going to hospital—only about 10% of parents complained that they were unable to schedule appointment with GP, nearly 90% of our patients were appointed straight to our Emergency Department. All patients admitted to our ED had COVID-19 Antigen Rapid Test, also protective measures in surgery preparation for COVID patients were not causing substantial delay in performing appendectomy. We noticed a statistically significant increase in the number of cases with peritonitis during the pandemic. Similar results were presented by Baral et al., and Fonseca et al. who observed a 6% and 15% increase in the complications of appendicitis in the form of perforation during lock-down period^7,8^. Results presented by Baral et al. contrary to our observations did not show statistical significance^8^. According to observations made by Reichert et al. the differences in the course of all surgery emergencies could be observed between pre-pandemic and pandemic period^9^. They observed an increase in the number of all severe septic abdominal complications during the pandemic^9^.

Moreover, Baral et al. during the pandemic period performed appendectomy only with the classical technique due to the cost, but also due to technical difficulties (necessity to use additional personal protection)^8^. Although during the pandemic year 2020, our department did not change the protocols of treatment of appendicitis—non-complicated cases of appendicitis were operated on laparoscopically, complicated cases were treated with open appendectomy, we noticed divergence in the type of surgical approach used during appendectomy. Laparoscopic appendectomy was performed more frequently before the COVID period. In the pandemic year 2020, there was also double increase in the number of conversion from the laparoscopic approach to the classic open surgery. Similar observations were done by Cano-Valderrama et al. who reported “a reduction in the proportion of patients undergoing a laparoscopic approach 63.6% vs. 43.3%”^10^. Moreover in our series of patients in 2020 the amount of cases threated with appendectomy and drainage increased substantially. However the differences in the white blood count and CRP serum levels in our patients with appendicitis treated in the year 2019 vs 2020 were nonsignificant^11^. Data provided by a multinational survey performed by WSES (World Society of Emergency Surgery) members also prove that the differences in the course and treatment of emergency surgeries were caused by higher number of complicated cases treated during the pandemic^9^.

In consequence, during the pandemic the duration of hospitalization was statistically longer as well. The length of hospitalization increased by almost a day (4.761 in 2019 vs 5.634 in 2020) among our patients. Delayed admission to the hospital, lack of the proper diagnosis at the early stage and, in consequence, late appendectomy, resulted in higher percentage of complicated cases of appendicitis in children treated in the year 2020^5^.

In contrast, in a study by Turanli et al. the length of hospitalization in patients with complicated and uncomplicated appendicitis was comparable, despite the increase in the number of complicated cases during the pandemic^6^.

The observed differences might have various explanations although one comes to the forefront—the COVID-19 pandemic which significantly redefined our reality. The closure of General Practitioner’s offices in the first throes of 2020 could explain the delay in seeking help in hospital. The acute abdominal pain—the primary symptom of appendicitis, might resemble many other ailments, and proper diagnosis requires physical examination which was impossible with only phone contact with a doctor. Still according to some authors, the delay was not caused by surgical team. Stöß et al. in their survey proved that emergency operations were performed without restrictions or delay although their number decreased by 30%^12^.

Another important consequence of pandemic was associating healthcare workers as the potential source of contracting the novel infectious disease. Many patients have stayed at home till the very last moment due to fear of contact with medical staff who was overexposed to COVID patients. Lazzerini et al. in their survey showed that caregivers of pediatric patients delayed their visit to Emergency Department due to fear of COVID-19 infection^13^. We noted significantly longer time between the onset of symptoms, and presentation to the hospital in patients admitted during pandemic. Still among our patients in the pandemic year 2020, only two cases were COVID positive. Also according to study by Cano-Valderrama et al. only small number of emergency patients—0.75% was found to be COVID positive^10^. In our Department the number of children operated on because of appendicitis in the pandemic year was smaller than in the previous year, but the rate of complicated cases was higher, probably reflecting the delay in ED presentation. The same observation was done by Fahrner et al.^2^. At last we should stress the consequences of obesity among children—a potential factor influencing the course of the disease, even though we did not notice significant difference in BMI of our pediatric patients treated with appendectomy in 2019 and 2020. The obesity, which was definitely also a problem before the pandemic with a rising trend, has inevitably enlarged its scale due to lockdown, cutting off children from many forms of physical activity.

Our results definitely prove the unfavourable effect of the SARS Cov-2 pandemic on the pediatric surgery practice. Similar observations were also previously reported^14^. We hope that our study can change the approach of patients, medical staff and Health Care System with the aim to accelerate the proper diagnosis, and decrease the rate of complications connected with appendicitis.

The data that support the findings of this study are available on request from the corresponding author EM.

## Conclusions

Fear of being infected, the limited availability of appointments at General Practitioners and the new organization of the medical health care system during pandemic, delay proper diagnosis of appendicitis. Aforementioned delay leads to higher number of complicated cases, treated with open appendectomy and drainage of abdominal cavity, higher number of conversions from the laparoscopic to classic open technique, and longer hospitalization of children treated with appendectomy in the year of pandemic, which also substantially rise the costs of health-care system.
